# Complete genome sequence of *Nakamurella multipartita* type strain (Y-104^T^)

**DOI:** 10.4056/sigs.721316

**Published:** 2010-03-30

**Authors:** Hope Tice, Shanmugam Mayilraj, David Sims, Alla Lapidus, Matt Nolan, Susan Lucas, Tijana Glavina Del Rio, Alex Copeland, Jan-Fang Cheng, Linda Meincke, David Bruce, Lynne Goodwin, Sam Pitluck, Natalia Ivanova, Konstantinos Mavromatis, Galina Ovchinnikova, Amrita Pati, Amy Chen, Krishna Palaniappan, Miriam Land, Loren Hauser, Yun-Juan Chang, Cynthia D. Jeffries, John C. Detter, Thomas Brettin, Manfred Rohde, Markus Göker, Jim Bristow, Jonathan A. Eisen, Victor Markowitz, Philip Hugenholtz, Nikos C. Kyrpides, Hans-Peter Klenk, Feng Chen

**Affiliations:** 1 DOE Joint Genome Institute, Walnut Creek, California, USA; 2 DSMZ - German Collection of Microorganisms and Cell Cultures GmbH, Braunschweig, Germany; 3 MTCC - Microbial Type Culture Collection, Institute of Microbial Technology, Chandigarh, India; 4 Los Alamos National Laboratory, Bioscience Division, Los Alamos, New Mexico, USA; 5 Biological Data Management and Technology Center, Lawrence Berkeley National Laboratory, Berkeley, California, USA; 6 Oak Ridge National Laboratory, Oak Ridge, Tennessee, USA; 7 HZI – Helmholtz Centre for Infection Research, Braunschweig, Germany; 8 University of California Davis Genome Center, Davis, California, USA

**Keywords:** polysaccharide-accumulating, septa-forming, nonmotile, Gram-positive, MK-8 (H_4_), ‘*Microsphaeraceae*’, *Frankineae*, GEBA

## Abstract

*Nakamurella multipartita* (Yoshimi *et al*. 1996) Tao *et al*. 2004 is the type species of the monospecific genus *Nakamurella* in the actinobacterial suborder *Frankineae*. The nonmotile, coccus-shaped strain was isolated from activated sludge acclimated with sugar-containing synthetic wastewater, and is capable of accumulating large amounts of polysaccharides in its cells. Here we describe the features of the organism, together with the complete genome sequence and annotation. This is the first complete genome sequence of a member of the family *Nakamurellaceae*. The 6,060,298 bp long single replicon genome with its 5415 protein-coding and 56 RNA genes is part of the *** G****enomic* *** E****ncyclopedia of* *** B****acteria and* *** A****rchaea * project.

## Introduction

Strain Y-104^T^ [1] (DSM 44233 = ATCC 700099 = JCM 9533) is the type strain of the species *Nakamurella multipartita*, which is the sole member and type species of the genus *Nakamurella* [2], the type genus of the family *Nacamurellaceae* [2]. *N. multipartita* was first described in 1996 by Yoshimi *et al*. as polysaccharide-accumulating ‘*Microsphaera multipartita*’ and type species of the genus ‘*Microsphaera*’ [1]. Unfortunately, Yoshimi *et al*. [1] overlooked the priority of the named fungal genus *Microsphaera* described 145 years earlier [3]. Principle 1(2) of the *International Code of Nomenclature of Bacteria* (1990 Revision) recommends avoiding the use of names which might cause confusion and therefore grants priority of the fungal genus *Microsphaera* in the family *Erysiphaceae* [4], Stackebrandt *et al*. maintained the illegitimate name when creating the likewise illegitimate family ‘*Microsphaeraceae*’ in 1997 [5]. In 2004 Tao *et al*. replaced the illegitimate genus and family names with the legitimate and validly published names *Nakamurella* and *Nakamurellaceae*, respectively, in honor of the Japanese microbiologist Kazonuri Nakamura, who also discovered strain Y-104^T^ [2]. Here we present a summary classification and a set of features for *N. multipartita* strain Y-104^T^, together with the description of the complete genomic sequencing and annotation.

## Classification and features of organism

The environmental diversity of the members of the species *N. multipartita* appears to be limited. Only one 16S rRNA gene sequence from a Finish indoor isolate (BF0001B070, 96.2% sequence identity) is reported in Genbank [6], as well as two Finish indoor phylotypes (FM872655, 98.2%; FM873571, 96.2%) by Taubel *et al*., and a phylotype from fresh water sediment of the high altitude Andean Altiplano (northern Chile) with 96.6% sequence identity (EF632902). None of the sequences generated from large scale environmental samplings and genome surveys surpassed 93% sequence identity and were thereby significantly less similar to strain Y-104^T^ than the closest related type strain, DS-52 ^T^ of *Humicoccus flavidus* (95.9%) [7] (status November 2009).

**Figure 1:**
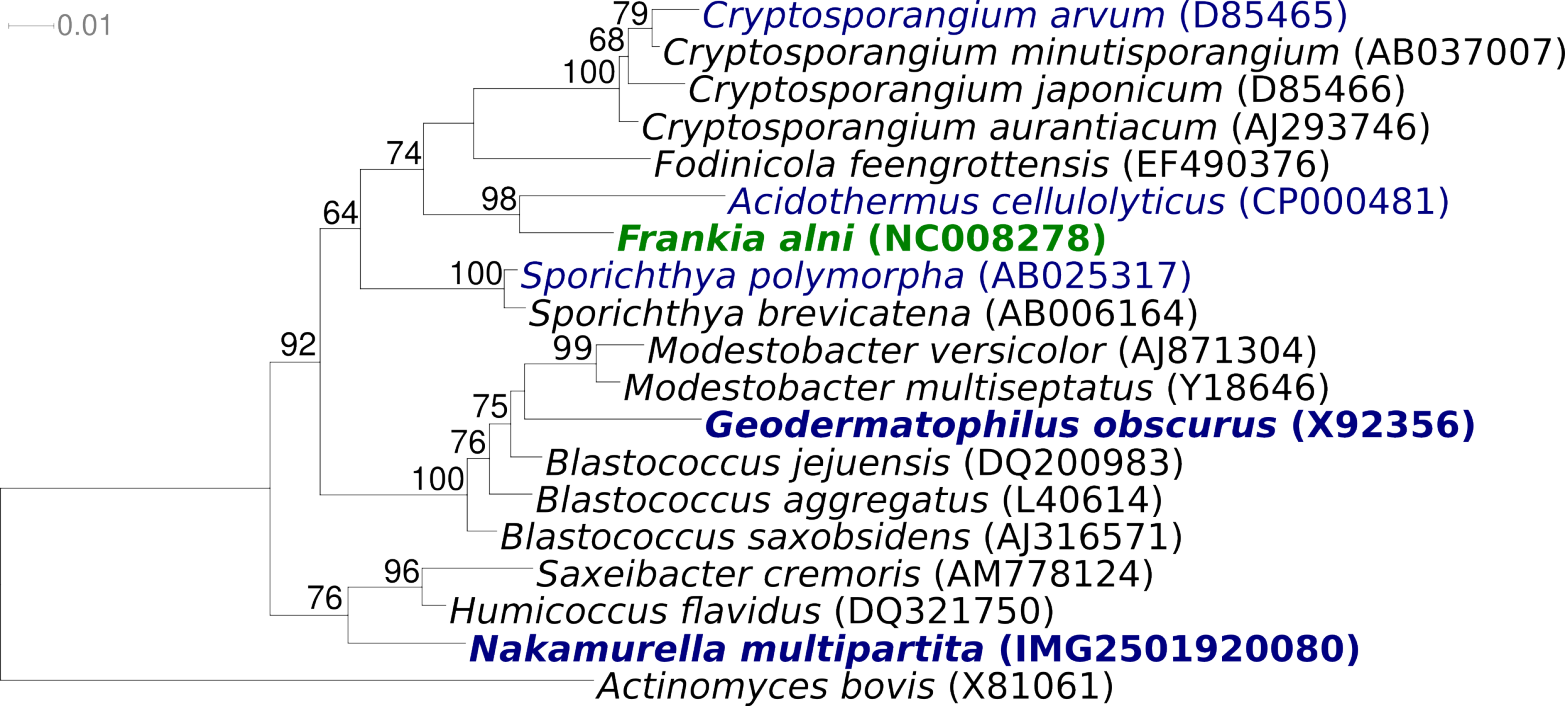
Phylogenetic tree highlighting the position of *N. multipartita* Y-104^T^ relative to the other type strains within the *Frankineae*. The tree was inferred from 1362 aligned characters [8,9] of the 16S rRNA gene sequence under the maximum likelihood criterion [10] and rooted with the type strain of the order. The branches are scaled in terms of the expected number of substitutions per site. Numbers above branches are support values from 1,000 bootstrap replicates if larger than 60%. Lineages with type strain genome sequencing projects registered in GOLD [11] such as the GEBA organism *Geodermatophilus obscurus* [12] are shown in blue. Important non-type strains are shown in green [13], and published genomes in bold.

Figure 1 shows the phylogenetic neighborhood of *N. multipartita* strain Y-104^T^ in a 16S rRNA based tree. The sequences of the two identical 16S rRNA gene copies differ by one nucleotide (C-homopolymer close to 3’-end) from the previously published 16S rRNA sequence generated from JCM 9543 (Y08541).

**Figure 2:**
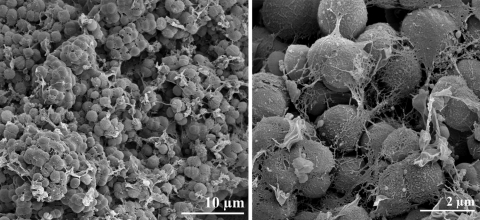
Scanning electron micrograph of *N. multipartita* strain Y-104^T^

*N. multipartita* strain Y-104^T^ is aerobic and chemoorganotrophic. Cells are non-motile, non-spore forming, Gram-positive (Table 1) and coccus-shaped [1]. The cells are 0.8 to 3.0 µm in diameter; depending on the growth stage. They occur as singles, in pairs or in small irregular clusters (Figure 2). A rod-coccus cycle was not observed at any stage of the growth. Strain Y-104^T^ has a characteristic cell division in which a cell wall-like structure occurs in the middle of each cell during their early growth phase. Such structures, also called septa, were frequently observed during the late log phase of the growth cycle [1]. The doubling time was reported to be approximately 11 hours in a liquid medium at pH 7.0 and at 25°C [1]. Colonies on agar plates are circular, smooth, convex and white at the early stage of growth and cream-colored at later stage of growth. The polysaccharide content of the cells is very high, sometimes more than 50% (wt/wt) depending on the culture conditions.

**Table 1:** Classification and general features of *N. multipartita* strain Y-104^T^ according to the MIGS recommendations [14]

**MIGS ID**	**Property**	**Term**	**Evidence code**
	Current classification	Domain *Bacteria* Phylum *Actinobacteria* Class *Actinobacteria* Order *Actinomycetales* Suborder *Frankineae* Family *Nakamurellaceae* Genus *Nakamurella* Species *Nakamurella multipartita* Type strain Y-104	TAS [15] TAS [16] TAS [5] TAS [5] TAS [2] TAS [2] TAS [2] TAS [2] TAS [1]
	Gram stain	positive	TAS [1]
	Cell shape	coccus	TAS [1]
	Motility	non-motile	TAS [1]
	Sporulation	non-sporulating	TAS [1]
	Temperature range	10-35°C	TAS [1]
	Optimum temperature	25°C	TAS [1]
	Salinity	up to 6g NaCl/L	TAS [1]
MIGS-22	Oxygen requirement	aerobic chemoorganotroph	TAS [1]
	Carbon source	sugars, alcohols, glucose, maltose, mannose, fructose, starch	TAS [1]
	Energy source	starch, ethanol, propanol	TAS [1]
MIGS-6	Habitat	activated sludge cultured in fed-batch reactors	TAS [1]
MIGS-15	Biotic relationship	free-living	NAS
MIGS-14	Pathogenicity	none	NAS
	Biosafety level	1	TAS [17]
	Isolation	activated sludge	TAS [1]
MIGS-4	Geographic location	not reported	
MIGS-5	Sample collection time	not reported	
MIGS-4.1 MIGS-4.2	Latitude Longitude	not reported	
MIGS-4.3	Depth	not reported	
MIGS-4.4	Altitude	not reported	

Evidence codes - IDA: Inferred from Direct Assay (first time in publication); TAS: Traceable Author Statement (i.e., a direct report exists in the literature); NAS: Non-traceable Author Statement (i.e., not directly observed for the living, isolated sample, but based on a generally accepted property for the species, or anecdotal evidence). These evidence codes are from of the Gene Ontology project [18]. If the evidence code is IDA, then the property was directly observed for a live isolate by one of the authors, or an expert mentioned in the acknowledgements.

Growth of strain Y-104^T^ occurs at a temperature range of 10-35˚C and a pH range of 5.0 to 9.0 and in the presence of up to 6% NaCl. *N. multipartita* is positive for catalase production and negative for oxidase activity [1]. It is capable of utilizing glucose, fructose, mannose, galactose, xylose, sucrose, maltose, lactose, mannitol, sorbitol, ethanol, propanol, glycerol, starch, pyruvate, aranine, glutamate, glutamine and histidine as carbon and energy sources [1]. The strain cannot utilize acetate, malate, succinate, arginine, asparagine, methanol or glycogen as carbon and energy sources [1]. Strain Y-104^T^ is able to accumulate large amounts of polysaccharides in its cells [1].

### Chemotaxonomy

 The murein of *N. multipartita* strain Y-104^T^ contains *meso*-diaminopimelic acid as the diagnostic diamino acid [1]. The fatty acid pattern of Y-104^T^ is dominated by iso-C_16:0_ (19.7%), iso-C_15:0_ (15.7%) and C_18:1_ (14.0%) and substantial amounts of C_16:0_ (10.3%), anteiso-C_15:0_ (9.2%), iso-C_17:0_ (8.5%) and anteiso-C_17:0_ (5.2%) were detected [1]. The predominant menaquinones are MK-8 (H_4_), approximately 97.0%, and minor amounts of MK-7 (H_4_), MK-8 (H_2_) and MK-9 (H_4_) were present [1]. Mycolic acids are absent [1].

## Genome sequencing and annotation

### Genome project history

This organism was selected for sequencing on the basis of each phylogenetic position, and is part of the *** G****enomic* *** E****ncyclopedia of* *** B****acteria and* *** A****rchaea * project. The genome project is deposited in the Genome OnLine Database [14] and the complete genome sequence is deposited in GenBank. Sequencing, finishing and annotation were performed by the DOE Joint Genome Institute (JGI). A summary of the project information is shown in Table 2.

**Table 2:** Genome sequencing project information

**MIGS ID**	**Property**	**Term**
MIGS-31	Finishing quality	Finished
MIGS-28	Libraries used	Two Sanger genomic libraries: 8kb pMCL200 and fosmid pcc1Fos
MIGS-29	Sequencing platforms	ABI3730
MIGS-31.2	Sequencing coverage	15.4× Sanger
MIGS-30	Assemblers	Arachne, phrap
MIGS-32	Gene calling method	Prodigal, GenePRIMP
	INSDC ID	CP001737
	Genbank Date of Release	September 18, 2009
	GOLD ID	Gi02230
	NCBI project ID	29537
	Database: IMG-GEBA	2501939634
MIGS-13	Source material identifier	DSM 44233
	Project relevance	Tree of Life, GEBA

### Growth conditions and DNA isolation

*N. multipartita* Y-104^T^, DSM 44233, was grown in DSMZ 553 medium [19] at 28°C. DNA was isolated from 1-1.5 g of cell paste using Qiagen Genomic 500 DNA Kit (Qiagen, Hilden, Germany) following the manufacturer's instructions with modification st/FT for cell lysis according to Wu *et al*. [20].

### Genome sequencing and assembly

The genome was sequenced using Sanger sequencing platform. All general aspects of library construction and sequencing can be found on the JGI website. Optimal raft assembly was produced using Arachne assembler. Finishing assemblies were made using the parallel phrap assembler (High Performance Software, LLC). Possible mis-assemblies were corrected with Dupfinisher [21] or transposon bombing of bridging clones (Epicentre Biotechnologies, Madison, WI). Gaps between contigs were closed by editing in Consed, custom primer walk or PCR amplification. A total of 2,596 Sanger finishing reads were produced to close gaps, to resolve repetitive regions, and to raise the quality of the finished sequence. The error rate of the completed genome sequence is less than 1 in 100,000. Together all Sanger reads provided 15.4× coverage of the genome. The final assembly contains 118,931 Sanger reads.

### Genome annotation

Genes were identified using Prodigal [22] as part of the Oak Ridge National Laboratory genome annotation pipeline, followed by a round of manual curation using the JGI GenePrimp pipeline [23]. The predicted CDSs were translated and used to search the National Center for Biotechnology Information (NCBI) nonredundant database, UniProt, TIGRFam, Pfam, PRIAM, KEGG, COG, and InterPro databases. Additional gene prediction analysis and manual functional annotation was performed within the Integrated Microbial Genomes - Expert Review (IMG-ER) platform [24].

## Genome properties

**Figure 3:**
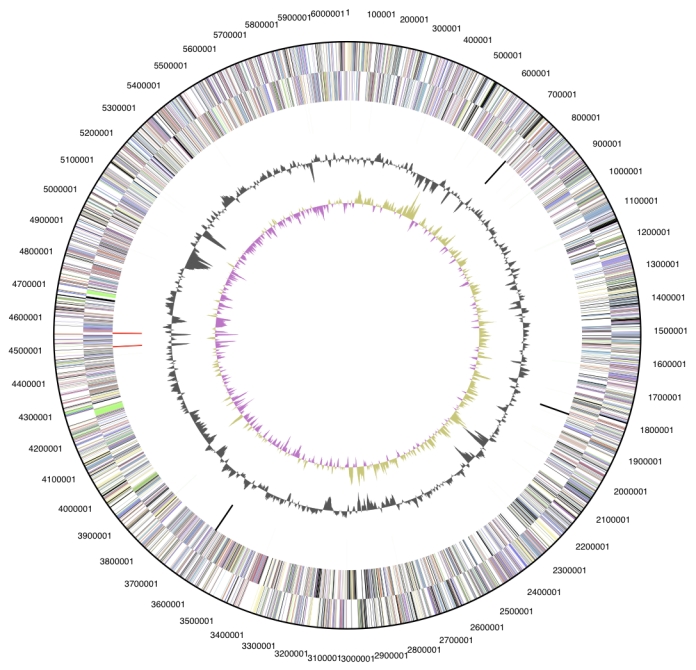
Graphical circular map of the genome. From outside to the center: Genes on forward strand (color by COG categories), Genes on reverse strand (color by COG categories), RNA genes (tRNAs green, rRNAs red, other RNAs black), GC content, GC skew.

The genome is 6,060,298 bp long and comprises one main circular chromosome with a 70.9% G+C content (Table 3 and Figure 3). Of the 5,471 genes predicted, 5,415 were protein coding genes, and 56 RNAs; 175 pseudo genes were also identified. The majority of the protein-coding genes (66.5%) were assigned a putative function while the remaining ones were annotated as hypothetical proteins. The distribution of genes into COGs functional categories is presented in Table 4.

**Table 3:** Genome Statistics

**Attribute**	**Value**	**% of Total**
Genome size (bp)	6,060,298	100.00%
DNA coding region (bp)	5,526,464	91.19%
DNA G+C content (bp)	4,297,749	70.92%
Number of replicons	1	
Extrachromosomal elements	0	
Total genes	5,471	100.00%
RNA genes	56	1.02%
rRNA operons	2	
Protein-coding genes	5,415	98.98%
Pseudo genes	175	3.20%
Genes with function prediction	3,638	66.50%
Genes in paralog clusters	3,319	60.67%
Genes assigned to COGs	3,673	67.14%
Genes assigned Pfam domains	4,054	74.10%
Genes with signal peptides	1,713	31.31%
Genes with transmembrane helices	1,258	22.99%
CRISPR repeats	9	

**Table 4:** Number of genes associated with the general COG functional categories

**Code**	**Value**	**%age**	**Description**
J	160	3.9	Translation, ribosomal structure and biogenesis
A	2	0.0	RNA processing and modification
K	400	9.7	Transcription
L	324	7.8	Replication, recombination and repair
D	31	0.8	Cell cycle control, mitosis and meiosis
V	81	2.0	Defense mechanisms
T	238	5.8	Signal transduction mechanisms
M	173	4.2	Cell wall/membrane biogenesis
Z	1	0.0	Cytoskeleton
U	44	1.1	Intracellular trafficking and secretion
O	113	2.7	Posttranslational modification, protein turnover, chaperones
C	308	7.5	Energy production and conversion
G	341	8.3	Carbohydrate transport and metabolism
E	334	8.1	Amino acid transport and metabolism
F	97	2.4	Nucleotide transport and metabolism
H	190	4.6	Coenzyme transport and metabolism
I	160	3.9	Lipid transport and metabolism
P	182	4.4	Inorganic ion transport and metabolism
Q	117	2.8	Secondary metabolites biosynthesis, transport and catabolism
R	506	12.2	General function prediction only
S	330	8.0	Function unknown
-	1773	32.4	Not in COGs
